# Menstrual characteristics, menstrual anxiety and school attendance among adolescents in Uganda: a longitudinal study

**DOI:** 10.1186/s12905-021-01544-6

**Published:** 2021-12-11

**Authors:** Clare Tanton, Kevin Nakuya, Catherine Kansiime, Laura Hytti, Belen Torondel, Suzanna C. Francis, Prossy Namirembe, Shamirah Nakalema, Ruth Nalugya, Saidat Namuli Musoke, Stella Neema, David A. Ross, Chris Bonell, Janet Seeley, Helen A. Weiss

**Affiliations:** 1 Faculty of Public Health and Policy, London School of Hygiene & Tropical Medicine, 15-17 Tavistock Place, London, WC1H 9SH UK; 2 Medical Research Council/Uganda Virus Research Institute & London School of Hygiene & Tropical Medicine Uganda Research Unit, Entebbe, Uganda; 3 WoMena Uganda, Kampala, Uganda; 4 Faculty of Infectious and Tropical Diseases, London School of Hygiene and Tropical Medicine, London, UK; 5 MRC International Statistics & Epidemiology Group, Faculty of Epidemiology and Population Health, London School of Hygiene and Tropical Medicine, London, UK; 6 Uganda Virus Research Institute, Entebbe, Uganda; 7 College of Humanities and Social Science, Makerere University, Kampala, Uganda; 8 Department of Infectious Disease Epidemiology, London School of Hygiene and Tropical Medicine, London, UK

## Abstract

**Background:**

Qualitative data show negative impacts of menstruation on health and education in many settings, but there are few longitudinal quantitative studies of the impact of menstruation. We analyse associations with menstrual anxiety and school attendance in a study of Ugandan secondary school students.

**Methods:**

Data were from a longitudinal pilot study of a menstrual health intervention (MENISCUS), conducted in two secondary schools in Entebbe sub-district, Uganda. Self-completed menstrual-related data, including menstrual anxiety, were collected from 232 participants pre- and post-intervention. A sub-cohort of 100 randomly-selected post-menarcheal girls were asked to self-complete daily diaries during 10 months of follow-up, with data on menstrual flow, pain, and school attendance. We used multivariable logistic regression to estimate associations with menstrual anxiety among all girls at baseline, and random-effects logistic regression to estimate associations of menstrual characteristics with school non-attendance for 3 months pre-intervention in the sub-cohort, adjusting for within-girl clustering.

**Results:**

Overall, 130/222 (58.6%) of menstruating girls reported being anxious about their next period. Menstrual anxiety was higher in those not living with their mother (adjusted odds ratio (OR) = 1.91; 95% confidence interval (CI) 1.01–3.60), believing menstrual myths (aOR = 1.83; 0.95–3.50 for not agreeing that it is healthy for a girl to run, dance or cycle during her period; aOR = 1.97; 1.04–3.73 for agreeing that when a girl has her period she is unclean), lower menstrual confidence (aOR = 2.49; 1.33–4.65 for avoiding physical activity during her period; aOR = 1.68; 0.89–3.17 for not feeling comfortable to talk to other girls about her period; aOR = 2.89; 1.28–6.54 for agreeing that boys/girls tease them about their periods; and aOR = 2.27; 1.21–4.27 for worrying about being teased during her period). Those with lower knowledge about menstruation were less likely to report anxiety (aOR = 0.44; 0.23–0.84). During the pre-intervention period of the sub-cohort, school non-attendance was associated with menstrual pain, with 21.7% of girls missing school on days when they reported pain vs. 8.3% on days when no pain was reported (aOR = 3.82; 1.66–8.77).

**Conclusions:**

Menstruation causes substantial anxiety in Ugandan schoolgirls, and menstrual pain is associated with missing school on period-days. Menstrual health interventions should address socio-cultural aspects of menstruation to reduce anxiety, and provide education on pain management strategies to support school attendance.

**Supplementary Information:**

The online version contains supplementary material available at 10.1186/s12905-021-01544-6.

## Background

Menstruation is frequently experienced in a negative way, impacting adversely on mental and physical health [1, 2], education [3–5] and employment [6]. A recent systematic review and qualitative meta-synthesis of menstrual experiences in 76 studies in low- and middle-income countries (LMICs) [6] proposed a directional model of the menstrual experience in which the socio-cultural context (including menstrual stigma and gender norms) impact on the menstrual experience by limiting social support, shaping behavioural expectations and limiting knowledge about menstruation [6]. The menstrual experience is also affected by (i) scarce physical and economic resources through limiting access to menstrual materials and the infrastructure required to support menstruation and (ii) individual menstrual factors (such as pain and flow) [6]. Menstrual fear and worry were highlighted across these qualitative studies as representing a significant burden to the lives of women and girls, with anxiety related to managing menstruation [7–9]. We are not aware of any quantitative data exploring menstrual anxiety.

Most quantitative research describing menstrual cycles in girls has been cross-sectional [10–18]. Many of these studies (from India, Nigeria, Egypt, Italy, Jordan, Ethiopia, Ghana and Morocco) focused on menstrual irregularity, although some studies also reported on menstrual pain. Menstrual pain was reported by 38% of female secondary school students in Jordan, and of those reporting pain, 8% reported needing to miss at least one day of school per menstrual cycle [11]. Longitudinal studies of menstrual cycles have focused on those with menstrual cycle disorders [19, 20], and there are few longitudinal quantitative data which document variability in menstrual cycles and characteristics in the general population, or associations with school attendance in adolescents. A systematic review of quantitative studies found associations between menstrual practices and both school attendance and performance in cross sectional studies [2] and several menstrual health (MH) interventions have been developed with an aim of improving school attendance [1, 21, 22]. A better understanding of the menstrual characteristics (e.g. flow, pain, day of menstruation) associated with school attendance would assist in the design of such interventions to improve school attendance, performance and broader health and wellbeing for girls and young women.

The aim of this paper is to understand characteristics of menstruation and examine associations with menstrual anxiety and school attendance in Ugandan secondary school students, using data from a recently-completed pilot trial of a school-based MH intervention [22, 23]. This paper has three objectives—to describe (1) factors associated with participants reporting feeling anxious about their next menstrual period at baseline; (2) the characteristics of participants’ menstruation (including flow and pain, how they vary within and between girls, and menstrual factors associated with pain) over 10 months; and (3) the relationship between menstrual cycle characteristics and school/class attendance over the first 3 months (pre-intervention).

## Methods

Data were collected as part of a longitudinal pilot trial of a multi-component school-based MH intervention (“MENISCUS”) conducted in two secondary schools in the Entebbe Municipality in Uganda. The aim of the parent study was to pilot test the MENISCUS intervention to prepare for a future cluster-randomised trial which will evaluate impact on secondary school performance and attendance, mental health and well-being, and menstrual knowledge, practices and self-efficacy, in Uganda[22]. Eligible participants were girls and boys in the second year of school (S2). The intervention was delivered over 6 months (February to July 2018). The informed adolescent’s assent and parent/guardian’s written informed consent was obtained prior to engaging students in the research activities. Adolescents withdrawing assent and/or without written permission from parents/guardians were excluded from the study.

### Study design

**Fig. 1:**
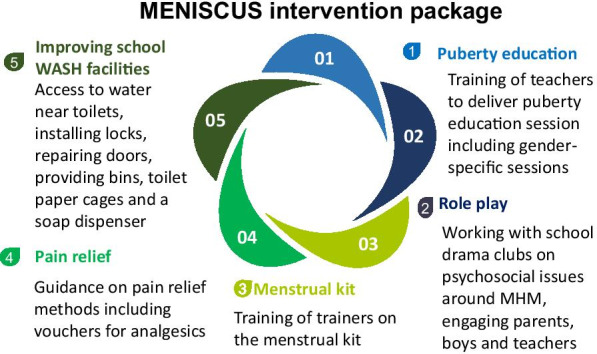
MENISCUS intervention package

The parent study was prospective, with pre-post evaluation of the MENISCUS intervention. The intervention is shown in Fig. 1. Full details of the design and results of the evaluation have been reported [22, 23]. All participants self-completed a questionnaire at baseline (October 2017) and endline (August 2018) on tablet computers using OpenDataKit software. At both surveys, information was collected on socio-demographics, knowledge of puberty, menstruation and pain management methods, menstrual myths, perceptions of menstruation, including menstrual anxiety, menstrual management practices at last menstrual period (LMP) and experiences of pain and leakage at LMP and behavioural and conduct problems using the 25-item Strengths and Difficulties questionnaire (SDQ) [24]. An additional file shows the measures from the baseline survey which were used in this study to answer objective 1 (Additional file 1). In addition, a sample of 50 female students per school were randomly chosen from those self-reporting having started menstruating in the baseline questionnaire, to participate in a nested cohort and self-complete daily diaries throughout the 10 months of follow-up. Diaries were provided for every term and holiday and participants were asked to record on each day whether they were menstruating and to self-define the flow as light, moderate or heavy, whether they had ‘period pain’, as well as school and class attendance. Data on pain, school and class attendance were collected each day, irrespective of whether participants were menstruating that day. An additional file shows the diary developed for the study which was self-completed by female students (Additional file 2).

Data for the current analyses focus on menstrual experience in the absence of an intervention, so we analyse baseline survey data from the parent study (objective 1), the 10 months of longitudinal data from the diaries to characterise menstrual patterns and characteristics (objective 2); and the 3 months pre-intervention data from the diaries to assess associations of menstrual characteristics with school attendance (objective 3).

### Data management

Survey data captured electronically were stored on the LSHTM ODK secure server and transferred to Stata 15 for analysis. Diary data were paper-based and were double-entered using Microsoft Access and transferred to Stata 15 for data management and analysis.

### Statistical analyses

**Fig. 2:**
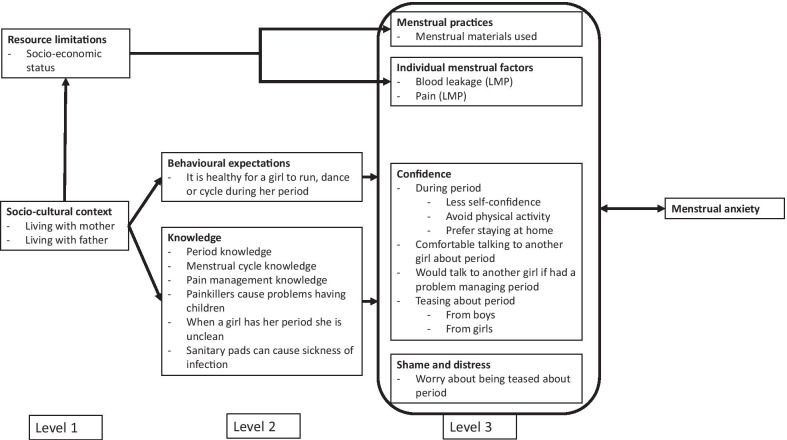
Proposed model for menstrual anxiety, drawing on figure two in [6]

Data from the baseline survey were used to examine the association between reported anxiety about next menstrual period (outcome) and menstrual factors (exposure) using logistic regression (objective 1). Measures are described in Additional file 1. Anxiety about next menstrual period was defined according to participants stating that they agreed or strongly agreed with the following statement: “I feel anxious about having my next period”. Using the Hennegan model of menstrual experience [6], we conceptualised anxiety about the next menstrual period as representing an aspect of menstrual confidence, and therefore a core part of the menstrual experience (Fig. 2). We first examined associations of anxiety with each variable. Then, aligned with the Hennegan model, we examined the most distal antecedents (level 1) i.e. those related to resource limitations (socio-economic status) and socio-cultural context (school, ethnic group, religion, living with mother, living with father), and retained factors independently associated with anxiety (using a cut-off *p* < 0.1). We created a similar model for the next level of antecedents (level 2), which relate to behavioural expectations (disagreement with the statement ‘it is healthy for a girl to run, dance or cycle during her period’) and knowledge and beliefs around puberty and menstruation, retaining factors from this level independently associated with anxiety (*p* < 0.1). We then created a model assessing the association between anxiety and variables related to menstrual practices, individual menstrual factors, confidence around menstruation, and shame and distress around menstruation (level 3), retaining factors independently associated (*p* < 0.1). We present multivariable models containing (a) level 1 and 2 variables (model A); and (b) level 1, 2 and 3 variables (model B). In all analyses we allowed for clustering by school by including school as a fixed effect.

Diary data were used to describe menstrual cycles during the 10 months of follow-up (objective 2). Initial analyses were at menstrual period-level, to estimate menstrual patterns (mean duration of cycle: number of days between the start of one menstrual period and the start of the next menstrual period; mean duration of menstruation (with standard deviation (SD)); perceived menstrual flow; and pain. Repeated-measures linear regression with robust standard errors was used to compare duration of menstrual cycle and menstrual period, by age group (14–16; 17–19 years) and years since starting menstruation (< 3; ≥ 3 years). To capture differences in participants’ experience of menstruation, the following participant-level summary statistics (summarised over all menstrual cycles per participant) were calculated: (i) percentage of days of menstruation with self-reported heavy flow and (ii) percentage of days of menstruation with pain. Further analyses were at day-level, adjusting for clustering by participant using random-effects logistic regression to estimate odds ratios (OR) and 95% confidence intervals (CI) for the association between pain (outcome variable) with flow (light/medium/heavy) and day of menstrual period (1st day vs. not 1st day).

The relationship between menstrual cycle characteristics and school/class attendance (objective 3) was analysed using the diary data collected during the first term of data collection (October-December 2017) i.e. prior to implementation of the intervention, which began in February 2018. Non-school days (i.e. weekends and school holidays) were excluded. Outcomes were (i) school attendance (attended a full day of school—including if they missed some classes) and (ii) class attendance (i.e. attended a full day at school and all classes). Random-effects logistic regression (adjusting for within-participant clustering) was used to estimate ORs and 95%CI for associations of each outcome with: day of menstrual period (1st day, not 1st day); menstrual flow (light, moderate, heavy) and pain (yes/no).

## Results

Of the 252 S2 female students at baseline, 232 (92%) consented/assented. At baseline, the mean age was 15.4 years (SD 1.31; range 12–20 years). The majority (n = 193; 83.2%) were Christian and 93 (42.7%) were of Ganda ethnicity. Relatively few participants (n = 33; 14.2%) had running water at home, and 30 (12.9%) had a flush toilet in the home.

### Factors associated with anxiety about next period

Overall, 222 girls (95.7%) reported that they had started menstruating at baseline and were included in analyses of menstrual anxiety. At baseline, over half (58.6%; 130/222) of participants reported being anxious about their next period. Table 1 shows associations with menstrual anxiety. There was no evidence of an association between anxiety about the next period and socio-economic status or living with their father. However, girls who did not live with their mother were more likely to report being anxious about their next period (68.1% vs. 48.6% of girls living with their mothers; OR = 2.25; 95% CI 1.30–3.90).

**Table 1:** Factors associated with feeling anxious about next period

		Anxious about next period		Unadjusted		Model A—variables from levels 1 and 2		Model B—variables from levels 1, 2 and 3	
	Total	No.	%	OR	95% CI	aOR	95% CI	aOR	95% CI
Level 1									
*Resource limitations*									
Socio-economic status					*p* = 0.42				
Higher SES	116	71	61.2	1					
Lower SES	106	59	55.7	0.80	(0.47–1.37)				
*Socio-cultural context*									
School									
School A						1		1	
School B						1.14	(0.61–2.15)	1.13	(0.57–2.26)
Ethnic group					*p* = 0.18				
Muganda	102	58	56.9	1					
Munyankole	34	25	73.5	2.07	(0.86–4.97)				
Other	86	47	54.7	0.91	(0.51–1.63)				
Religion					*p* = 0.84				
Roman Catholic	77	48	62.3	1					
Anglican	52	28	53.8	0.72	(0.35–1.47)				
Other Christian	54	32	59.3	0.88	(0.43–1.79)				
Muslim	38	22	57.9	0.88	(0.39–2.01)				
Lives with mother					*p* < 0.01		*p* = 0.03		*p* = 0.05
Yes	109	53	48.6	1		1		1	
No	113	77	68.1	2.25	(1.30–3.91)	1.87	(1.05–3.36)	1.91	(1.01–3.60)
Lives with father					*p* = 0.65				
Yes	85	37	43.5	1					
No	137	55	40.1	1.14	(0.66–1.97)				
Level 2									
*Behavioural expectations*									
It is healthy for a girl to run, dance or cycle during her period					*p* = 0.03		*p* < 0.01		*p* = 0.07
Agrees	107	55	51.4	1		1		1	
Doesn't agree	115	75	65.2	1.81	(1.05–3.12)	2.27	(1.26–4.11)	1.83	(0.95–3.50)
*Menstrual knowledge*									
No. menstrual biology questions correct^a^					*p* = 0.02		*p* < 0.01		*p* = 0.01
≤ 7	133	69	51.9	0.50	(0.28–0.88)	0.46	(0.25–0.85)	0.44	(0.23–0.84)
8/9	89	61	68.5	1		1		1	
No. menstrual cycle questions correct^b^					*p* = 0.92				
0/1	79	46	58.2	1					
2/3	143	84	58.7	1.03	(0.59–1.80)				
No. effective pain management methods known^c^					*p* = 0.24				
0–1 methods	99	58	58.6	1.42	(0.73–2.74)				
2–3 methods	64	42	65.6	1.87	(0.90–3.87)				
4+ methods	59	30	50.8	1					
Painkillers cause problems having children					*p* = 0.80				
Disagrees	126	73	57.9	1					
Doesn't disagree	96	57	59.4	1.07	(0.62–1.84)				
When a girl has her period she is unclean					*p* < 0.01		*p* < 0.01		*p* = 0.04
Disagrees	116	57	49.1	1		1		1	
Doesn't disagree	106	73	68.9	2.32	(1.34–4.02)	2.50	(1.38–4.51)	1.97	(1.04–3.73)
Sanitary pads can cause sickness or infection					*p* = 0.31				
Disagrees	141	79	56.0	1					
Doesn't disagree	81	51	63.0	1.34	(0.76–2.35)				
Level 3									
*Menstrual practices*									
Only used manufactured materials at LMP^d^					*p* = 0.14				
Yes	162	90	55.6	1					
No	60	40	66.7	1.59	(0.85–2.96)				
*Individual menstrual factors*									
Blood leaked through clothes in LMP					*p* = 0.57				
No	82	50	61	1					
Yes	74	48	64.9	0.83	(0.43–1.59)				
Pain during LMP					*p* = 0.65				
No	57	32	56.1	1					
Yes	165	98	59.4	0.87	(0.47–1.60)				
*Menstrual confidence*									
During my period I feel less self-confident than during other days					*p* < 0.01				
Disagrees	51	21	41.2	1					
Doesn't disagree	171	109	63.7	2.50	(1.31–4.76)				
During my period I avoid physical activity (e.g. walking, running)					*p* < 0.01				*p* < 0.01
Disagrees	110	50	45.5	1				1	
Doesn't disagree	112	80	71.4	3.04	(1.74–5.32)			2.49	(1.33–4.65)
I prefer staying at home during my period rather than going to school					*p* < 0.01				
Disagrees	167	89	53.3	1					
Doesn't disagree	55	41	74.5	2.64	(1.33–5.23)				
I feel comfortable to talk to other girls at school about my period					*p* = 0.07				*p* = 0.11
Agrees	103	54	52.4	1				1	
Doesn't agree	119	76	63.9	1.67	(0.97–2.89)			1.68	(0.89–3.17)
If I had a problem with managing my period, I would talk to another girl about it					*p* = 0.93				
Agrees	163	96	58.9	1					
Doesn't agree	59	34	57.6	0.97	(0.53–1.79)				
Boys/girls tease me about my period					*p* < 0.01				*p* = 0.01
Disagrees	171	90	52.6	1				1	
Doesn't disagree	51	40	78.4	3.25	(1.56–6.77)			2.89	(1.28–6.54)
*Menstrual shame and distress*									
I worry about being teased during my period					*p* < 0.01				*p* = 0.01
Disagrees	95	43	45.3	1				1	
Doesn't disagree	127	87	68.5	2.62	(1.51–4.54)			2.27	(1.21–4.27)

^a^Menstruation knowledge statements (response true/false): Adolescence is the time between puberty and menstruation; changes in the body during puberty happen because of hormones; the physical changes related to puberty usually start between 10 and 14 years of age in girls, and between 12 and 16 in boys; menstrual blood comes from the stomach where food is digested; women stop menstruating after the age of about 40–50; menstruation in girls and women is normal; pregnant women menstruate; when a girl gets her first period, her body is ready to have children; during her period a girl can get pregnant

^b^Menstrual cycle questions (closed responses): what is period blood; how long does a period usually last; how many days are there usually between periods

^c^Effective methods: painkiller, drinking water, using hot water bottle, exercise, relaxing, foods with lots of water

^d^Reusable or disposable pads, tampons or menstrual cups

Anxiety about the next period was more common among those who disagreed that it is healthy for a girl to run, dance or cycle during her period (65.2% vs. 51.4%) and among those who agreed that when a girl has her period she is unclean (63.9% vs. 49.1%). Anxiety was less common among participants with poorer knowledge about menstrual biology (51.9% among those with ≤ 7/9 questions answered correctly vs. 68.5% among those with 8/9 questions answered correctly). These variables remained associated with anxiety after adjustment for each other and whether girls lived with their mother (Model A). There was no evidence of an association between anxiety and knowledge of the menstrual cycle, effective pain management methods or agreement that painkillers cause problems having children or sanitary pads can cause sickness or infection (Table 1).

With respect to the level 3 variables, there was no evidence that anxiety about the next period was associated with reporting using manufactured materials (i.e. reusable or disposable pads, tampons or menstrual cups) during the last menstrual period (LMP), blood leaking through clothes at LMP or pain during LMP. However, anxiety was associated with variables that may represent lower confidence around menstruation. Those agreeing with the following statements were more likely to be anxious: during my period I feel less confident than on other days (63.7% vs. 41.2%); during my period I avoid doing physical activity (71.4% vs. 45.5%); I prefer staying at home during my period rather than going to school (74.5% vs. 53.3%); boys/girls tease me about my period (78.4% vs. 52.6%). Girls who didn’t report feeling comfortable talking to other girls at school about their period (63.9% vs. 52.4%) or reporting that they worry about being teased during their period were also more likely to report feeling anxious (68.5% vs. 45.3%). In a model containing all level 3 variables, girls who agreed with the following statements were more likely to be anxious: during my period I avoid doing physical activity and boys/girls tease me about my period. Girls reporting that they do not feel comfortable talking to another girl about their period or who reported worrying about being teased during their period were also more likely to report feeling anxious.

In the multivariable model including variables from all levels (Model B), the following factors were associated with being more likely to report anxiety about their next period: not living with their mother (aOR = 1.91 [1.01–3.60]), not agreeing that it is healthy for a girl to run, dance or cycle during her period (aOR = 1.83 [0.95–3.50]), agreeing that when a girl has her period she is unclean (aOR = 1.97 [1.04–3.73]), avoiding physical activity during her period (aOR = 2.49 [1.33–4.65]), boys/girls teasing her during her period (aOR = 2.89 [1.28–6.54]) and worrying about being teased during her period (aOR = 2.27 [1.21–4.27]). Those with lower general knowledge about menstruation biology were less likely to report anxiety about their next period (aOR = 0.44 [0.23–0.84]). The association between avoiding physical activity and anxiety was weaker in this model adjusted for level 1 and 2 variables suggesting some confounding by these variables. Associations between disagreeing that it is healthy for a girl to run, dance or cycle during her period and not disagreeing that when a girl has her period she is unclean were also attenuated in the final model (Model B) suggesting that they were mediated through the level 3 variables.

The Hennegan model suggests that menstrual experience impacts on psychological health. In our study, participants who reported being anxious about their next period had a higher mean SDQ score than those who did not report being anxious (11.0 (SD5.7) vs. 9.1 (SD5.5)) but there was no association after adjustment for level 1, 2 and 3 variables in the final model (aOR = 1.02, 95%CI 0.96–1.08) implying that they have similar causal factors.

### Menstrual characteristics during follow-up

#### Summary of sample for diary data

All 100 post-menarcheal girls selected for the diary sub-study agreed to participate. Diaries were collected at least once from 99/100 girls, with complete diary data for 9 months of follow-up (6 diaries) available for 77 (77%) girls. Of the 22 girls with incomplete diary data, 13 submitted data from one diary, three from two diaries, three from three diaries and two from four diaries, although these diaries were not all complete. Missing diaries were due to students leaving schools rather than dropping out of diary completion. The mean age at enrolment was 16.5 years (SD 1.19; range 14–19 years). The median age at menarche was 13 (IQR 12–14) and median years since menarche was 3 (IQR 2–4).

#### Duration of menstrual periods and menstrual cycles

**Fig. 3:**
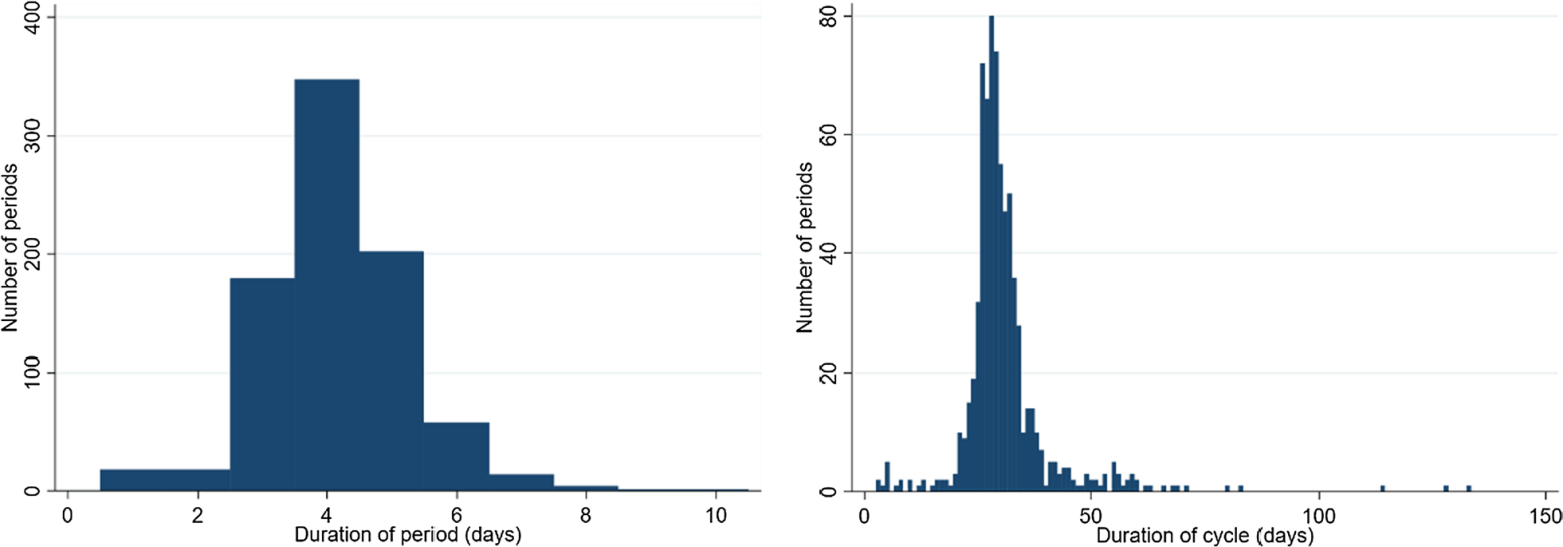
Distribution of lengths of 842 menstrual periods (left) and 739 menstrual cycles (right) reported by 99 girls

A total of 842 menstrual periods were reported by the 99 girls with diary data. The reported mean length of these menstrual periods was 4.1 days (SD 1.1 days; median 4 days [IQR 3–5 days]; Fig. 3) with no evidence, after allowing for within-participant clustering, of a difference by age (14–16 years vs. 17–20 years: 4.1 vs. 4.2 days; *p* = 0.67) or by years since menarche (< 3 vs. 3 + : 4.2 vs 4.1 days; *p* = 0.48). Overall, 739 complete menstrual cycles were reported by 94 of the 99 girls completing the diaries (i.e. those with data collected for at least 2 periods). The mean duration of menstrual cycles was 30.8 days (SD 10.5 days; median 29 days [IQR 27–32]; Fig. 3). There was no evidence, after allowing for within-participant clustering, of a difference by age (31.1 vs. 30.4 days; *p* = 0.88) or by years since menarche (30.8 vs. 30.8 days; *p* = 0.94).

#### Menstrual flow and menstrual pain

**Fig. 4:**
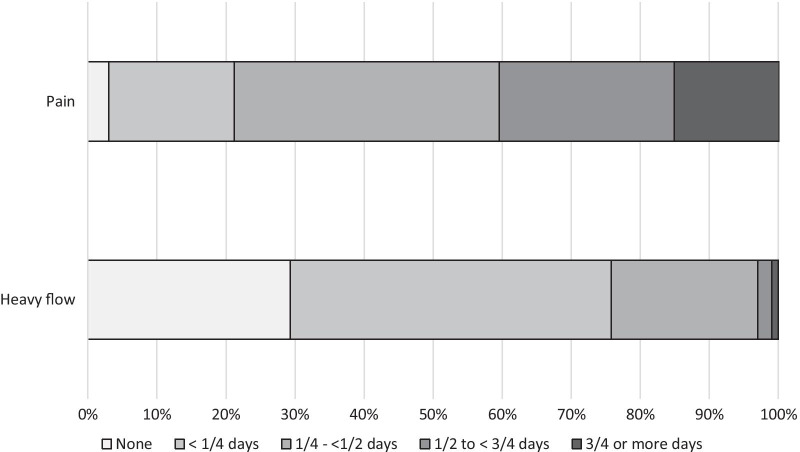
Participant-level experience of menstruation characteristics

For most menstrual periods (61.3%; 516/842), participants reported no days of heavy flow. One day of heavy flow was reported for 22.0% of periods (185/842) and more than one day of heavy flow for 16.4% of periods (138/842). Participants were more likely to report a light flow on the first day of their period (n = 517/842; 61.4%) than on subsequent days (n = 1052/2663; 39.5%; OR = 2.75 [2.32–3.27]). Overall, 70.7% of girls reported at least one heavy flow day, with 24.2% reporting heavy flow on at least one-quarter of period-days (Fig. 4). The proportion of girls reporting heavy flow was similar on first (110/842; 13.1%) and subsequent period days (388/2663; 14.6%; OR = 0.89 [0.70–1.14]).

Pain was reported during three-quarters of menstrual cycles (74.5%; 627/842), and on almost half of period-days (43.7%; 1532/3505). For one in six cycles (16.5%; 139/842), pain was reported on all period-days. Overall, few participants (3.0%; 3/99) reported no days of pain during follow-up, 18.2% reported pain on fewer than a quarter of period-days and 15.2% reported pain on at least three-quarters of period-days (Fig. 4). Pain was more likely to be reported on moderate or heavy flow days than on light flow days (35.2% [552/1569] reported pain on light flow days vs 45.3% on moderate flow days (OR = 1.56 [1.32–1.86] vs. light flow days, and 66.1% on heavy flow days (OR = 4.08 [3.17–5.27] vs. light flow days). Pain was more commonly reported on first period days (65.3%) vs. subsequent period days (36.9%; OR = 4.60 [3.80–5.57] for first vs. subsequent days). Period pain was also reported on 0.2% (51/21986) of all non-period days, most of which (69%; 35/51) were the day before the period began. In total, pain was reported on 4.2% of the days immediately before menses. There was no evidence that age or years since starting menstruation either confounded or modified these associations with the exception of the association between first vs subsequent period day and pain which was stronger in older girls (14–16 year olds: OR = 3.91 [3.05–5.02]; 15–17 year olds: OR = 5.70 [4.25–7.65]; p-value for interaction = 0.05).

### Menstrual patterns and school attendance

Using data from October-December 2017 (prior to the intervention), menstruation was associated with reduced school attendance. Participants reported not attending a full day of school on 15.8% of period-days (70/443) vs. 8.6% of non-period days (235/2736) (OR = 2.36 [1.72–3.23]). Table 2 shows the menstrual-related characteristics associated with missing school. On period-days, pain was strongly associated with not attending a full day of school with 21.7% of girls missing school on days when they reported pain vs. 8.3% on days when no pain was reported (OR = 4.22 [1.88–9.51]). There was weak evidence for an association between day of the period and not attending the full day of school with 20.7% girls reporting missing school on the first period-day versus 14.1% on subsequent period-days (OR = 1.83 [0.97–3.45]). There was no evidence of an association between heaviness of flow and school attendance (Table 2). After adjustment for all menstrual variables, pain remained strongly associated with school attendance (adjusted OR = 3.82 [1.66–8.77]) but there was no evidence of an association between first day and not attending a full day. Similar associations were seen with not attending all classes.

**Table 2:** Association between menstrual cycle characteristics and school attendance

	Total number of days	Not attending full school day^a^		
		Number (%) not attending full school days	Unadjusted OR (95%CI)	Adjusted OR (95%CI)^b^
*Pain*				
No	194	16 (8.3%)	1	1
Yes	249	54 (21.7%)	4.22 (1.88–9.51)	3.82 (1.66–8.77)
*First day of menstrual period*				
No	327	46 (14.1%)	1	1
Yes	116	24 (20.7%)	1.83 (0.97–3.45)	1.48 (0.72–3.06)
*Flow*				
Light	183	27 (14.8%)	1	1
Medium	197	33 (16.8%)	1.06 (0.54–2.06)	1.05 (0.49–2.24)
Heavy	63	10 (15.9%)	1.30 (0.49–3.43)	1.06 (0.37–3.02)

All odds ratios adjusted for school as a fixed effect

^a^Number of days on which the participant reported attending a full day of school—including if they didn’t attend all classes

^b^Adjusted for pain, day of period and flow

## Discussion

The study showed that menstrual anxiety in our study population of secondary school girls in Uganda is associated with not living with their mother, other markers of lack of menstrual confidence and lack of self-efficacy, and that menstrual pain is a key factor in causing school absence on period-days.

### Menstrual anxiety

The majority of participants in our study (58.6%) reported feeling anxious about their next period. These findings align with Hennegan’s meta-synthesis which reported that worry and fear featured prominently in women and girls’ menstrual experience [6]. In our study, menstrual anxiety was strongly associated with various aspects of the socio-cultural context, particularly not living with their mother, negative behavioural expectations, lack of menstrual confidence and shame (anxiety about being teased), possibly reflecting an internalised stigma. There was no evidence of an association with menstrual practices or individual menstrual factors (e.g. pain or blood leaking through clothes at last period), which may be due to a high proportion of this population reporting using manufactured menstrual materials during their last period.

We observed lower anxiety in girls living with their mothers, and those who reported feeling comfortable to talk to other girls at school about their period but no differences in anxiety according to whether girls were living with their fathers. Other studies have highlighted the challenges of sharing information about menstruation with fathers [8]. The association between menstrual anxiety and greater knowledge of menstrual biology is puzzling, but there was no evidence of an association between menstrual anxiety and knowledge about the menstrual cycle specifically and, reassuringly, no association between menstrual anxiety and greater knowledge of menstrual biology using data from the endline survey. Given the role boys play in teasing girls during their menstruation, also found in other studies [25, 26], interventions to improve girls’ menstrual experience should include both boys and girls to improve broad social support from the school community. We found an association between anxiety and a specific concern about being teased during menses but no evidence of an association of anxiety with either blood leaking through clothes during the last period, or with pain, despite the fact that in other studies teasing has been reported to be related to others knowing whether a girl was menstruating e.g. due to stains [4].

Our observed low levels of menstrual confidence among girls highlights the importance of addressing the socio-cultural context of menstruation. The importance of markers of social support and increased self-efficacy in managing anxiety around menstruation suggests that social cognitive theory (SCT) may be an appropriate framing for menstrual health interventions in schools [27]. SCT asserts that individuals learn by observing others, and that an observed behaviour is learnt and enacted if: (i) the individual has self-efficacy regarding the behaviour; (ii) individuals receive reinforcement for performing the behaviour; and (iii) the environment supports enactment. The theory suggests opportunities for providing social support, improving self-efficacy and using peer/observational learning to achieve behaviour change. After the pilot MENSICUS intervention, which is grounded in SCT, we found that the proportion of study girls reporting menstrual anxiety reduced from 58.6 to 34.4% [22]. However, there was evidence of persistent menstrual anxiety with about half of the participants who reported being anxious at baseline, also reporting anxiety at endline. Further research is needed to understand how best to address this.

### Impact of menstruation on school attendance

Access to quality education, especially for girls, is a key sustainable development goal set by the United Nations in 2016 [28]. In previous qualitative and cross-sectional studies, girls reported menstrual pain to be a major contributor to school absence [29–31]. By collecting prospective daily diary data, we were able to directly link school absence days with menstrual factors and examine the factors most strongly associated with school absence. Over the 10 months of follow-up, pain was reported on almost half of period-days, and with almost all participants (97.0%) reporting some menstrual pain during follow-up. Pain was more likely to be reported on days with moderate or heavy flow, and was the key factor associated with school absenteeism, as has been seen in previous studies [11, 13]. In our study, 1 in 5 girls reported missing school on period days when they reported pain, compared with 1 in 12 girls missing school on period days when no pain was reported. Further, we observed that the level of non-attendance on period days where there was no pain was similar to the level on non-period days, and after adjustment for pain there was no association between first day of the period and non-attendance suggesting that this association was mediated through pain. This finding highlights the need for improved education on pain management for adolescents, including addressing myths about use of painkillers and teaching alternative pain mitigation techniques such as stretching, exercise and use of hot-water bottles.

Previous studies show inconsistent evidence for a relationship between menstruation and school absenteeism [1, 26, 32–37]. This may be due to differences in the association by setting, and also due to challenges in documenting school attendance itself, and documenting associations with menstrual characteristics. Prospectively-collected diaries work well to link data on menstruation directly with school attendance. As reported previously [22], we validated the prospective daily diary entry of attendance with unannounced observation of attendance (“spot-checks”). Girls were seen on 328/330 (99.4%) of days when their diary stated they were present and were not seen on all 37 days when the diary stated that they were absent. School registers were incomplete and rarely completed by teachers in this setting. However, spot-checks are not a ‘gold standard’ for school attendance as girls may not be visible (e.g. in the bathroom) or may attend for only part of a day (this would not be captured). The parent MENISCUS-2 study found that girls were more likely to attend school during menstruation at endline than at baseline, with reasons including the training on pain management, tracking their menstrual cycle, and having reusable pads [22].

### Study limitations

A limitation of our study is the relatively small number of participants with detailed menstrual cycle data (n = 99). However, the multiple datapoints per participant resulted in good power to detect associations of menstrual factors with school attendance. Further, this was a random sample of all post-menarcheal girls in the study, with a high response and retention rate, and was unlikely to have suffered substantially from selection bias due to high levels of participation. Girls were provided with information about how to manage menstrual pain during the study so this might have affected the reporting of pain in the diaries. In our study we asked girls about period pain generally and we do not know the site of the pain. For example, previous studies have reported headache as an important menstrual symptom, particularly as part of the pre-menstrual syndrome [12, 13]. We do not know whether girls in our study would have considered headache as period pain and reported it. We were also unable to describe the association between detailed menstrual characteristics (e.g. percentage of menstrual days with pain or percentage of menstrual days with heavy flow) and menstrual anxiety since the detailed menstrual data were only available for the 99 girls who participated in the diary sub-study. We therefore used experiences during the last menstrual period as an imperfect proxy for this. Finally, our sample of two schools limits the external validity of our results, and meant that we were unable to look at associations between structural factors like school-based WASH facilities because we recruited from only two schools.

## Conclusions

Our analysis highlights the prevalence of menstrual anxiety and the key role of menstrual pain on girls’ education in our study in Uganda. The association between pain and missing school or individual classes suggests that menstrual health interventions in schools should include pain management. Further, to improve girls’ menstrual experience, it is essential to address the psychosocial aspects of menstruation in future interventions, in addition to the physical and biological aspects such as the provision of basic knowledge related to menstruation, access to menstrual hygiene products or access to WASH.

## Supplementary Information

**Additional file 1**. Measures used in the paper. A table of measures used in the paper

**Additional file 2**. Daily diary. An image of the diary self-completed by participants

## Data Availability

Data are available on reasonable request. Data will be made available in the LSHTM Data Compass repository on request from the corresponding author (Clare Tanton https://orcid.org/0000-0002-4612-1858) from the website https://datacompass.lshtm.ac.uk/.
