# Supplementary material for: Menstrual characteristics, menstrual anxiety and school attendance among adolescents in Uganda: a longitudinal study
Source: BMC Womens Health. 2021 Dec 11;21:410. doi: 10.1186/s12905-021-01544-6 (PMC8665501; doi:10.1186/s12905-021-01544-6)
Supplement: Supplementary file 1 — Additional file 1. Measures used in the paper. A table of measures used in the paper [file 12905_2021_1544_MOESM1_ESM.pdf]

Supplementary file 1: Measures used in the analysis of factors associated with feeling anxious about the next period (objective 1) and how these relate to the model (Figure 2)

LEVEL	Variable	Definition of measure used in analysis	How the variable was measured
	**Baseline survey**		
**1: Resource limitations**	Socio-economic status	Positive responses were added to give a total of 0-9. A binary variable was then created splitting the sample into approximately half.<br>Higher SES – score of 3-9<br>Lower SES – score of 0-2	Participants were asked whether their household had each of the following items:<br>- Electricity<br>- Running water inside house<br>- Refrigerator<br>- TV<br>- More than 10 chickens or other birds<br>- Cows, goats, sheep, rabbits or pigs<br>- Moving motorbike<br>- Moving car<br>- Land for farming
**2: Behavioural expectations**	It is healthy for a girl to run, dance or cycle during her period	Binary variable created:<br>1. Strongly agrees/agrees<br>2. Neither agrees or disagrees/disagrees/strongly disagrees	Participants were asked to what extent they agreed with the statement with response options:<br>Strongly agree, Agree, Neither agree nor disagree, Disagree or Strongly disagree
**2: Menstrual knowledge**	No. menstruation biology questions correct	Correct responses were summed to give a total score ranging from 0-9	Participants were asked whether each of the following statements were true or false:<br>- Adolescence is the time between puberty and adulthood.<br>- Changes in the body during puberty happen because of hormones.<br>- The physical changes related to puberty usually start between 10 and 14 years of age in girls,<br>- and between 12 and 16 in boys.<br>- Menstrual blood comes from the stomach where food is digested.<br>- Women stop menstruating after the age of about 45–50.<br>- Menstruation in girls and women is normal.<br>- Pregnant women menstruate.

			- When a girl gets her first period, her body is ready to have children.<br>- During her period a girl can get pregnant.
	No. menstrual cycle questions correct	Correct responses summed to give a total score ranging from 0-3.	Participants were asked each of the following questions with closed responses:<br>- What is period blood?<br>- How long does a period usually last?<br>- How many days are there usually between periods?
	No. effective pain management methods known	Correct responses are shown in bold. Correct responses were summed and a categorical variable was created based on whether girls knew:<br>0/1 method<br>2/3 methods<br>4+ methods	Participants were asked:<br>What are effective ways of managing period pains? Response options (select all that apply) were:<br>- Nothing<br>- **Painkillers**<br>- **Drinking lots of clean water**<br>- **Holding a warm water bottle on the stomach**<br>- **Exercise and stretching**<br>- **Relaxing**<br>- **Eating foods with lots of water, like watermelon**<br>- Drinking soda<br>- Eating spicy foods<br>- Taking antibiotics<br>- I don't know<br>- Other
	Painkillers cause problems having children	Binary variable created:<br>1. Strongly disagrees/disagrees<br>2. Neither agrees or disagrees/agrees/strongly agrees	Participants were asked to what extent they agreed with the statement with response options:<br>Strongly agree, Agree, Neither agree nor disagree, Disagree or Strongly disagree
	When a girl has per period she is unclean	Binary variable created:<br>1. Strongly disagrees/disagrees<br>2. Neither agrees or disagrees/agrees/strongly agrees	Participants were asked to what extent they agreed with the statement with response options:<br>Strongly agree, Agree, Neither agree nor disagree, Disagree or Strongly disagree

	Sanitary pads can cause sickness or infection	Binary variable created:<br>1. Strongly disagrees/disagrees<br>2. Neither agrees or disagrees/agrees/strongly agrees	Participants were asked to what extent they agreed with the statement with response options:<br>Strongly agree, Agree, Neither agree nor disagree, Disagree or Strongly disagree
**3: Menstrual practices**	Only used manufactured methods at last menstrual period	Manufactured methods are shown in bold. Those only reporting using these methods were a yes for this variable, all others were a no.	Participants were asked:<br>Which of the following did you use during your most recent period?<br>Response options (select all that apply) were:<br>- **Afripads or other re-usable pad**<br>- **Always or other pad you can throw away**<br>- **Ruby Cup or other menstrual cup**<br>- Old clothes<br>- Toilet paper<br>- Cotton wool<br>- Knickers only<br>- Locally made pads you throw away e.g. made of banana fibres or other local materials<br>- **Tampons**<br>- New clothes<br>- Other
**3: Individual menstrual factors**	Blood leaked through clothes in last menstrual period	Binary variable.	Participants were asked:<br>Did blood leak through your clothes during your most recent period?<br>Yes<br>No
**3: Menstrual confidence**	Pain during last menstrual period	Binary variable.	Participants were asked:<br>Did you have any pain during your last period?<br>Yes<br>No
	During my period I feel less self-confident	Binary variable created:<br>1. Strongly disagrees/disagrees	Participants were asked to what extent they agreed with the statement with response options:<br>Strongly agree, Agree, Neither agree nor disagree, Disagree or Strongly disagree

	than during other days.	2. Neither agrees or disagrees/agrees/strongly agrees	
	During my period I avoid physical activity (eg, walking, running).	Binary variable created:<br>1. Strongly disagrees/disagrees<br>2. Neither agrees or disagrees/agrees/strongly agrees	Participants were asked to what extent they agreed with the statement with response options:<br>Strongly agree, Agree, Neither agree nor disagree, Disagree or Strongly disagree
	I prefer staying at home during my period rather than going to school.	Binary variable created:<br>1. Strongly disagrees/disagrees<br>2. Neither agrees or disagrees/agrees/strongly agrees	Participants were asked to what extent they agreed with the statement with response options:<br>Strongly agree, Agree, Neither agree nor disagree, Disagree or Strongly disagree
	I worry about being teased during my period.	Binary variable created:<br>1. Strongly disagrees/disagrees<br>2. Neither agrees or disagrees/agrees/strongly agrees	Participants were asked to what extent they agreed with the statement with response options:<br>Strongly agree, Agree, Neither agree nor disagree, Disagree or Strongly disagree
	I feel comfortable to talk to other girls at school about my period.	Binary variable created:<br>1. Strongly agrees/agrees<br>2. Neither agrees or disagrees/disagrees/strongly disagrees	Participants were asked to what extent they agreed with the statement with response options:<br>Strongly agree, Agree, Neither agree nor disagree, Disagree or Strongly disagree
	If I had a problem with managing my period, I would talk to another girl about it.	Binary variable created:<br>1. Strongly agrees/agrees<br>2. Neither agrees or disagrees/disagrees/strongly disagrees	Participants were asked to what extent they agreed with the statement with response options:<br>Strongly agree, Agree, Neither agree nor disagree, Disagree or Strongly disagree

	Boys/girls tease me about my period	Binary variable created:<br>1. Strongly disagrees/disagrees with both statements<br>2. Neither agrees or disagrees/agrees/strongly agrees with one or more statements	Participants were asked to what extent they agreed with the following two statements:<br>Boys tease me about my period<br>Girls tease me about my period<br><br>Response options:<br>Strongly agree, Agree, Neither agree nor disagree, Disagree or Strongly disagree
**3. Menstrual shame and distress**	I worry about being teased during my period	Binary variable created:<br>1. Strongly disagrees/disagrees<br>2. Neither agrees or disagrees/agrees/strongly agrees	Participants were asked to what extent they agreed with the statement with response options:<br>Strongly agree, Agree, Neither agree nor disagree, Disagree or Strongly disagree
