# Supplementary material for: Menstrual characteristics, menstrual anxiety and school attendance among adolescents in Uganda: a longitudinal study
Source: BMC Womens Health. 2021 Dec 11;21:410. doi: 10.1186/s12905-021-01544-6 (PMC8665501; doi:10.1186/s12905-021-01544-6)
Supplement: Supplementary file 2 — Additional file 2. Daily diary. An image of the diary self-completed by participants [file 12905_2021_1544_MOESM2_ESM.pdf]

Supplementary file 2: Daily diary

		Did you go to school today?				Did you attend class today?			Are you in your period today?				Do you have period pain today?	
Day	Date	Full Day	Half Day	No	Holiday	Yes	Some	None	No	Light period	Moderate period	Heavy period	No	Yes
Mon	31 Jul 17	☐	☐	☐	☐	☐	☐	☐	☐	☐	☐	☐	☐	☐
Tue	01 Aug 17	☐	☐	☐	☐	☐	☐	☐	☐	☐	☐	☐	☐	☐
Wed	02 Aug 17	☐	☐	☐	☐	☐	☐	☐	☐	☐	☐	☐	☐	☐
Thu	03 Aug 17	☐	☐	☐	☐	☐	☐	☐	☐	☐	☐	☐	☐	☐
Fri	04 Aug 17	☐	☐	☐	☐	☐	☐	☐	☐	☐	☐	☐	☐	☐
Sat	05 Aug 17	☐	☐	☐	☐	☐	☐	☐	☐	☐	☐	☐	☐	☐
Sun	06 Aug 17	☐	☐	☐	☐	☐	☐	☐	☐	☐	☐	☐	☐	☐

Footnotes:

Remember to tick one box per column on each day

- *Did you go to school today? Full day=Yes, for the whole day.*
  Half day = Yes for half a day, No = Did not attend school although it was open, Holiday = Including public holidays and weekends when you do not study
- *Did you attend class today? Yes = All classses, Some = some classes; None = No class attended*
- *Are you in your period today? No = I am not having my periods, Light period = light flow,*
  Moderate period = average blood flow, Heavy period=heavy blood flow
- *Do you have period pain today? Yes = your stomach or back aches due to period, No = your stomach or back not aching today due to period.*
